# Postmenopausal women with HIV have increased tooth loss

**DOI:** 10.1186/s12903-023-03744-y

**Published:** 2024-01-08

**Authors:** Sunil Wadhwa, Taylor R. Finn, Karolina Kister, Satoko Matsumura, Michael Levit, Anyelina Cantos, Jayesh Shah, Bruno Bohn, Evanthia Lalla, John T. Grbic, Ryan T. Demmer, Michael T. Yin

**Affiliations:** 1 Division of Orthodontics, Columbia University College of Dental Medicine, New York, NY USA; 2 Division of Oral and Maxillofacial Radiology, Columbia University College of Dental Medicine, New York, NY USA; 3 Division of Infectious Diseases, Columbia University College of Physicians and Surgeons, New York, NY USA; 4 Division of Epidemiology and Community Health, University of Minnesota School of Public Health, Minneapolis, MN USA; 5 Division of Periodontics, Columbia University College of Dental Medicine, New York, NY USA; 6 Division of Foundational Sciences, Columbia University College of Dental Medicine, New York, NY USA

**Keywords:** Periodontal disease, Bone biology, Computed tomography, Women’s health, Alveolar bone

## Abstract

**Background:**

With effective antiretroviral therapy, people with HIV (PWH) are living longer and aging; the majority of PWH in the United States are now over the age of 50 and in women have gone through the menopause transition. Menopause potentiates skeletal bone loss at the spine, hip, and radius in PWH. The alveolar bone which surronds the teeth is different than long bones because it is derived from the neural crest. However, few studies have assessed the oral health and alveolar bone in middle aged and older women with HIV. Therefore, the objective of this study was to evaluate periodontal disease and alveolar bone microarchitecture in postmenopausal women with HIV.

**Methods:**

135 self-reported postmenopausal women were recruited (59 HIV-, 76 HIV + on combination antiretroviral therapy with virological suppression) from a single academic center. The following parameters were measured: cytokine levels (IFN-γ, TNF-α, IL-1β, IL-2, IL-5, IL-6, IL-7, IL-8, IL-10, IL-12p70, IL-13, IL-17 A, OPG, and RANKL) in gingival crevicular fluid, bleeding on probing, probing depth, clinical attachment loss, number of teeth present, alveolar crestal height, and alveolar bone microarchitecture.

**Results:**

The mean age of participants was 57.04+/-6.25 years and a greater proportion of women with HIV were black/African American (HIV + 68.42%, HIV- 23.73%; p < 0.001). There was no significant difference in bleeding on probing (p = 0.17) and attachment loss (p = 0.39) between women who were HIV infected vs. HIV uninfected. Women with HIV had significantly higher RANKL expression in Gingival Crevicular Fluid (HIV + 3.80+/-3.19 pg/ul, HIV- 1.29+/-2.14 pg/ul ; p < 0.001), fewer teeth present (HIV + 17.75+/-7.62, HIV- 22.79+/-5.70; p < 0.001), ), lower trabecular number (HIV + 0.08+/-0.01, HIV- 0.09+/-0.02; p = 0.004) and greater trabecular separation (HIV + 9.23+/-3.11, HIV- 7.99+/-3.23; p = 0.04) compared to women without HIV that remained significant in multivariate logistic regression analysis in a sub-cohort after adjusting for age, race/ethnicity, smoking status, and diabetes.

**Conclusion:**

Postmenopausal women with HIV have deterioration of the alveolar trabecular bone microarchitecture that may contribute to greater tooth loss.

## Introduction

Prior to the advent of effective antiretroviral therapy (ART) used to treat HIV (human immunodeficiency virus), people with HIV (PWH) were at risk for greater periodontal disease severity compared to the general population. [1, 2] Proinflammatory cytokines, such as IL-1β, IL-6, and TNF-α, are associated with oral inflammation, periodontitis, and bone resorption, and have previously been found in higher abundance in PWH. [3] However, a review of the current literature indicates widespread use of ART has improved periodontal parameters in PWH, which now better match outcomes people without HIV. [4]

ART has allowed PWH to experience longer life expectancies. [5] With extended life come aging-related risk factors and comorbidities, such as bone loss. [6] According to data from the National Health and Nutrition Examination Survey 2017–2018, older women have a greater prevalence of bone loss and fractures in long bones compared to younger women and men. [7] This can be attributed to menopause and a decrease in estrogen. [8] It is unlclear what the role of estrogen loss during menopause plays on the jaw bone. For example, the risk of mandibular fracture does not increase with age in women [9] and the effect of menopause on the jaw bones appears to be site specific. In one study it was shown that the thickness of the cortical crestal bone was thinner in the posterior maxilla but not in the anterior mxailla, anterior mandible and posterior mandible in women over the age of 50 compared to women under the age of 50. [10]

Older PWH who experience menopause have been shown to have greater bone loss than the general population. [11] We previously found that postmenopausal women with HIV have lower bone mineral density than postmenopausal women without HIV, and greater longitudinal bone loss [12, 13] In a separate study, we confirmed that menopause and HIV infection are independently associated with lower bone mineral density and have an additive effect on the lumbar spine and total hip bone mineral density. [14] However the role of HIV infection and menopause on the Jaw bone microarchitecture is unknown.

A recent meta-analyis concluded that postmenopausal osteoporosis patients are more likely to suffer from markers of periodontal disease including increased clinicial attachment loss, increased pocket depth and increased bleeding on probing. [15] Since postemenopausal women with HIV have accelerated skeletal long bone loss, it may be possible that they also experience greater alveolar bone loss leading to increased severity of periodontal disease. Therfore, this study aims to evaluate alveolar bone microarchitecture and periodontal disease in the postmenopausal women with and without HIV.

## Materials and methods

### Study population

This study was approved by the Columbia University Irving Medical Center Institutional Review Board (IRB-AAA5233). Written informed consent was obtained from all study subjects. As part of an ongoing study examining the mandibular bone microarchitecture in PLWH. Our primary outcome was changes in Alveolar crestal height levels. Based upon our preliminary data [16], with a sample size of 120, we will have > 90% power to detect the observed effect size of a difference of 0.4 mm between HIV + and HIV- postmenopausal women in ACH. 135 patients were recruited from the dental clinic and Comprehensive Health Program clinic at Columbia University Irving Medical Center from September 2017 to December 2022; 76 were women with HIV and 59 without HIV. Inclusion criteria for the PWH cohort were: (a) self-reported menopause status, defined as the absence of menstrual bleeding for greater than 12 months; (b) 35–70 years old; (c) HIV-infected as defined by documentation of a positive antibody test or detectable HIV-1 RNA level any time prior to enrollment. In addition, women with HIV had to be on combination ART for at least one year with virological suppression, have a CD4 count > 100 cells/µL at time of enrollment, and no opportunistic infections within the last six months prior to enrollment.

Inclusion criteria for women without HIV were: (a) self-reported menopause status; (b) 35–70 years old; (c) a negative HIV antibody test. Exclusion criteria for both groups included: (a) current chemo- or immunotherapy; (b) antibiotic use in the preceding three months other than prophylaxis for opportunistic infections; (c) history of bisphosphonate or other osteoporosis therapy; (d) current oral contraceptive, hormone therapy (HT), or testosterone supplementation.

Blood samples were collected using serum separator tubes, separated into serum aliquots, stored at − 80 °C, then thawed and batch-analyzed at the Irving Columbia University Irving Medical Center Biomarker Laboratory. Circulating estrogen levels were measured by Estradiol ELISA (Siemens Cat# LKE21).

### Periodontal examination

A full-mouth periodontal examination was performed on all study participants by calibrated dental examiners using a UNC 15 probe. Probing depth (PD), clinical attachment level (CAL), and bleeding on probing (BOP) were recorded on all teeth excluding third molars at six sites per tooth: mesio-buccal, mid-buccal, disto-buccal, mesio-lingual, mid-lingual, and disto-lingual. Periodontal status was classified according to the Centers for Disease Control and Prevention/American Academy of Periodontology (CDC/AAP) definitions [17]: (1) no/mild periodontitis: neither “moderate” nor “severe” periodontitis; (2) moderate periodontitis: $\ge$2 interproximal sites with CAL $\ge$4 mm (not on same tooth) or $\ge$2 interproximal sites with PD $\ge$5 mm (not on same tooth); (3) severe periodontitis: $\ge$2 interproximal sites with CAL $\ge$6 mm (not on same tooth) and $\ge$1 interproximal site with PD $\ge$5 mm. BOP was recorded as present or absent. All missing teeth, excluding third molars, were recorded.

### Gingival crevicular fluid (GCF) collection

Gingival crevicular fluid (GCF) samples were collected from the distal site of six index teeth: two molars, two premolars, and two incisors. The selected teeth included the maxillary right first molar (#3), the maxillary left central incisor (#9), the maxillary left first premolar (#12), the mandibular left first molar (#19), the mandibular right central incisor (#25), and the mandibular right first premolar (#28). If any of these teeth were missing, the next most anterior tooth in the same quadrant was selected and recorded. Supragingival plaque was removed, and the gingiva was dried with cotton and an air syringe. Precut periopaper strips (Oraflow, Smithtown, NY, USA) were introduced into the periodontal pocket until mild resistance was felt, angled to meet the midpoint of the distal surface, and held in place for 30 s. The strips were then placed in a single microcentrifuge tube containing 500 µL of sterile phosphate buffered saline (0.02 M phosphate, 0.15 M NaCl, pH 7.5, containing 0.05% Tween 20 [PBST; Fisher Scientific Co., Fair Lawn, NJ, USA]) and the GCF was eluted by centrifugation.

### Inflammatory cytokine assays in GCF

Samples were assayed for GCF cytokines (IFN-γ, TNF-a, IL-1β, IL-2, IL-5, IL-6, IL-7, IL-8, IL-10, IL-12p70, IL-13, IL-17 A, OPG, and RANKL) in pg/ml and in duplicate at the Salimetrics SalivaLab (Carlsbad, CA) using an electrochemiluminescence method developed and validated for GCF by Salimetrics for all assays except OPG (abcam OPG ELISA Kit (ab100617)). Calibration curves were generated to determine analyte concentration using a mix of standards for assays run in multiplex (IL-1 beta, IL-6, IL-8, TNF-a, IFN-γ, IL-2, IL-7, IL-10, IL-12p70). The average coefficient of variation for all samples tested was < 15%. Sample test volume was 25 µL of GCF per determination.

### Intraoral radiographs

Study subjects were exposed to a full mouth series of up to 11 standardized intraoral radiographs (seven anterior periapical radiographs and four posterior bitewing radiographs), taken on the Progeny Preva Unfors-XI (Midmark Corporation, Lincolnshire, Illinois, USA) at 60 kV, 7.0 mA and time range 0.10–0.16 s at a 20 cm source-to-skin distance. Alveolar crestal height (ACH) is defined as the distance in millimeters between the cementoenamel junction (CEJ) and the most coronal part of the alveolar crest directly adjacent to the root surface along the long axis of the tooth, and measured according to published methods. [18] ACH was measured by blinded investigators in up to 24 teeth at two sites per tooth (mesial and distal), excluding third molars and canines. Whole-mouth mean ACH was calculated by averaging the ACH levels in all teeth measured as previously described. [19]

### Cone beam computed tomography (CBCT) acquisition

High resolution cone beam computed tomography (CBCT) images of the alveolar bone were obtained by a Planmeca ProMax 3D Classic CBCT scanner (Planmeca Inc., Hoffman Estates, Illinois, USA) at 84 kVp, 8 mA, and 15 s scan time. The manufacturer’s standard high-resolution scanning protocol was used to acquire an 80 × 42 × 68 mm region at a nominal isotropic resolution of 100 μm. Participants were positioned in the scanner and secured using a temporal bone support and chin rest to reduce motion artifacts, and instructed to occlude on the posterior dentition in the position that provided the best fit. The aim was to obtain maximum occlusion.

**Fig. 1:**
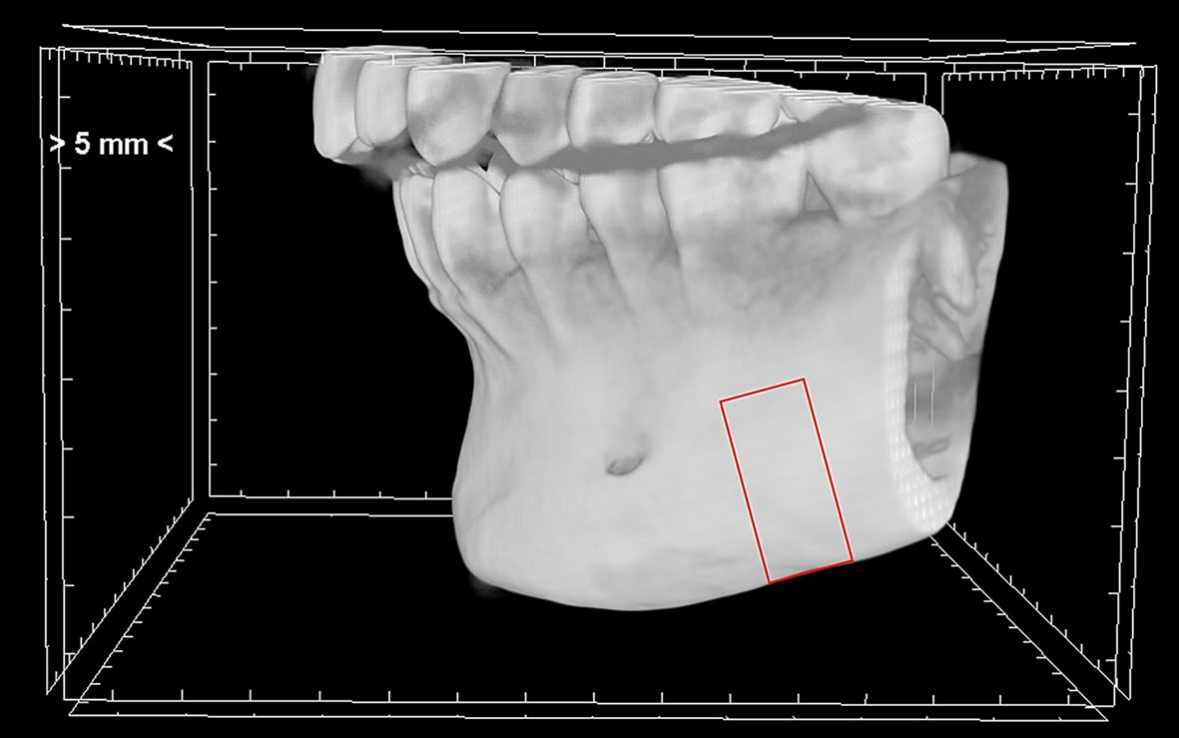
3-Dimensional cone beam reconstruction of lateral view of the mandible depicting the region of interest boundaries

To analyze the alveolar bone, 60 consecutive sections without intersection gaps were stacked after skipping the first 40 consecutive sections posterior to the opening of the mental foramen (Fig. 1). The region of interest included the trabecular and cortical bone, taken as the negative ROI from isolated trabecular bone. Skyscan Ctan Software (Bruker Corporation, Billerica, MA, USA) was used to isolate the ROI, convert to binary image form via local thresholding, and perform 3D microstructure evaluation. Parameters of interest included trabecular bone volume fraction (BV/TV), trabecular thickness, trabecular number, trabecular separation, cortical BV/TV, cortical thickness, and cortical porosity as previously described. [20]

### Statistical methods

Statistical analyses were conducted in R (4.2.2). Participant demographics and clinical characteristics were summarized for the study cohort and by HIV status. Variation in participant characteristics across HIV status were tested with F-statistics from type III ANOVA models or Chi Squared tests, as appropriate.

Univariable and multivariable linear regression models were used to investigate differences in odds of periodontitis across HIV status. All regression models were adjusted for participant age, race/ethnicity (black/Hispanic), smoking status, and history of type 2 diabetes. Adjusted analyses were only conducted in a subset of the cohort, excluding those with missing co variates and of white race, due to no HIV cases among participants who were white. We have complied with the STROBE guidelines for human observational studies.

## Results

The rationale of this cross sectional study was to examine periodontal disease activity and alveolar bone microarchitecture in postmenopausal women with and without HIV. A total of 135 postmenopausal women were recruited for the study (76 HIV+, 59 HIV-) with an average age of 57.04+/-6.25 years old (HIV + 56.95+/-5.06 yrs/old, HIV- 57.15+/-7.56 years/old; p = 0.85). Postmenopausal women with HIV had been on cART for a average of 17.79 +/- 7.4 years. There were significantly more black women (HIV + 68.42%, HIV-23.73%) and no white women (HIV + 0%, HIV- 20.34%) in the group with HIV (p < 0.001) (Table 1).

**Table 1:** Demographics, Gingival crevicular fluid biomarker cytokine levels, Periodontal and X -ray and CBCT Variables for the Full cohort of post-menopausal women with and without HIV. Biomarkers were performed for Interferon Gamma (IFNγ), Tumor Necrosis Factor Alpha (TNFα), Interleukin (IL) 1 alpha, 2, 5, 6, 7, 8, 10, 12p70, 13, and 17 alpha, Osteoprotegrin (OPG) and Receptor Activator of Nuclear Factor Kappa Beta (RANKL). Abbreviations used are Trabecular (Trab.), Bone Volume (BV), Total Volume (TV), Cortical (Cort.) and Alveolar Crestal Height (ACH)

Full Cohort					
Variable	# Missing	All	HIV -	HIV +	p-value
N		135	59	76	
Age	0	57.04 (6.25)	57.15 (7.56)	56.95 (5.06)	0.8507
Race/Ethn	0				**< 0.0001**
Black		66 (48.89%)	14 (23.73%)	52 (68.42%)	
Hispanic		57 (42.22%)	33 (55.93%)	24 (31.58%)	
White		12 (8.89%)	12 (20.34%)	0 (0%)	
Smoking	7				0.3730
No		90 (70.31%)	42 (72.41%)	48 (63.16%)	
Yes		38 (29.69%)	14 (24.14%)	24 (31.58%)	
Diabetes	5				0.1014
No		104 (80.0%)	49 (83.05%)	55 (72.37%)	
Yes		26 (20.00%)	7 (11.86%)	19 (25%)	
**Biomarkers** **pg/ml**	**# Not Detectable**				
Estadiol	20	38.29 (38.05)	43.0 (51.85)	35.16 (24.97)	0.2809
IFN g	23	1.42 (0.93)	1.44 (1.03)	1.4 (0.85)	0.8079
TNF a	20	1.32 (1.24)	1.35 (1.44)	1.3 (1.07)	0.8044
IL1 b	17	60.56 (65.58)	48.57 (56.31)	70 (71.05)	0.0780
IL2	20	0.56 (0.54)	0.5 (0.6)	0.6 (0.5)	0.3075
IL5	114	0.04 (0.03)	0.05 (0.05)	0.03 (0.01)	0.1627
IL6	21	0.71 (1.1)	0.54 (0.57)	0.85 (1.38)	0.1347
IL7	123	0.07 (0.05)	0.09 (0.05)	0.04 (0.02)	0.0749
IL8	17	586.87 (667.96)	514.26 (656.93)	644.07 (675.98)	0.2966
IL10	19	0.28 (0.25)	0.26 (0.24)	0.29 (0.25)	0.5288
IL12p70	75	0.26 (0.22)	0.31 (0.26)	0.23 (0.19)	0.2332
IL13	23	5.28 (3.35)	4.99 (3.58)	5.5 (3.18)	0.4245
IL17 a	56	0.58 (0.7)	0.6 (0.91)	0.57 (0.58)	0.8630
OPG	43	31.21 (28.55)	37.58 (42.88)	27.64 (15.12)	0.1099
RANKL	61	2.65 (3.02)	1.29 (2.14)	3.8 (3.19)	**0.0002**
**Periodontal Variables**	**# Missing**				
Periodontitis	8				0.9966
Mild		7 (5.51%)	3 (5.08%)	4 (5.26%)	
Moderate		45 (35.43%)	20 (33.90%)	25 (32.89%)	
Severe		75 (59.06%)	33 (55.93%)	42 (55.26%)	
Mean AL (mm)	10	3.49 (1.13)	3.58 (1.17)	3.41 (1.1)	0.3913
Mean PD (mm)	10	3.12 (0.92)	3.25 (1.01)	3.02 (0.83)	0.1657
% BOP	9	0.29 (0.26)	0.25 (0.23)	0.32 (0.28)	0.1680
# Teeth present	2	19.91 (7.28)	22.79 (5.7)	17.75 (7.62)	**< 0.0001**
**X-ray and CBCT Variables**	**# Missing**				
Trab BV/TV %	25	54.26 (15.74)	52.22 (16.32)	55.68 (15.29)	0.2596
Thickness (Pixels)	25	6.85 (1.57)	6.24 (1.78)	7.27 (1.25)	**0.0006**
Number (1/Pixels)	25	0.08 (0.02)	0.09 (0.02)	0.08 (0.01)	**0.0042**
Separation (Pixels)	25	8.73 (3.21)	7.99 (3.23)	9.23 (3.11)	**0.0450**
Cort BV/TV %	25	98.91 (1.59)	98.53 (1.8)	99.18 (1.38)	**0.0350**
Cort % Porosity	25	1.09 (1.59)	1.47 (1.8)	0.82 (1.38)	**0.0350**
Mean ACH (mm)	7	3.03 (1.12)	2.72 (1.01)	3.26 (1.28)	**0.0112**

### PWH have fewer teeth but similar periodontal disease activity

Postmenopausal women with HIV had significantly fewer teeth (HIV + 17.75+/-7.62 teeth, HIV- 22.79+/-5.70 teeth; p < 0.001) than postmenopausal women without HIV, with a maximum of 28 teeth present, excluding third molars. However, there was no significant differences in mean PD, CAL, or % BOP between HIV groups (Table 1).

### PWH have increased GCF markers of bone resorption

GCF levels of IFN-γ, TNF-a, IL-1β, IL-2, IL-5, IL-6, IL-7, IL-8, IL-10, IL-12p70, IL-13, IL-17 A (pg/ml), and OPG were similar in the two groups. GCF RANKL expression was significantly higher in women with HIV (HIV + 3.80+/-3.19 pg/ml, HIV- 1.29+/-2.14 pg/ml; p = 0.0002) (Table 1).

### PWH have increased alveolar bone loss and microcrhitectural alterations

Two-dimensional intraoral radiographs revealed that mean ACH was greater in women with HIV (HIV + 3.26+/-1.28 mm, HIV- 2.72+/-1.01 mm; p = 0.01) than women without HIV, where higher values indicate greater alveolar bone loss (Table 1).

**Fig. 2:**
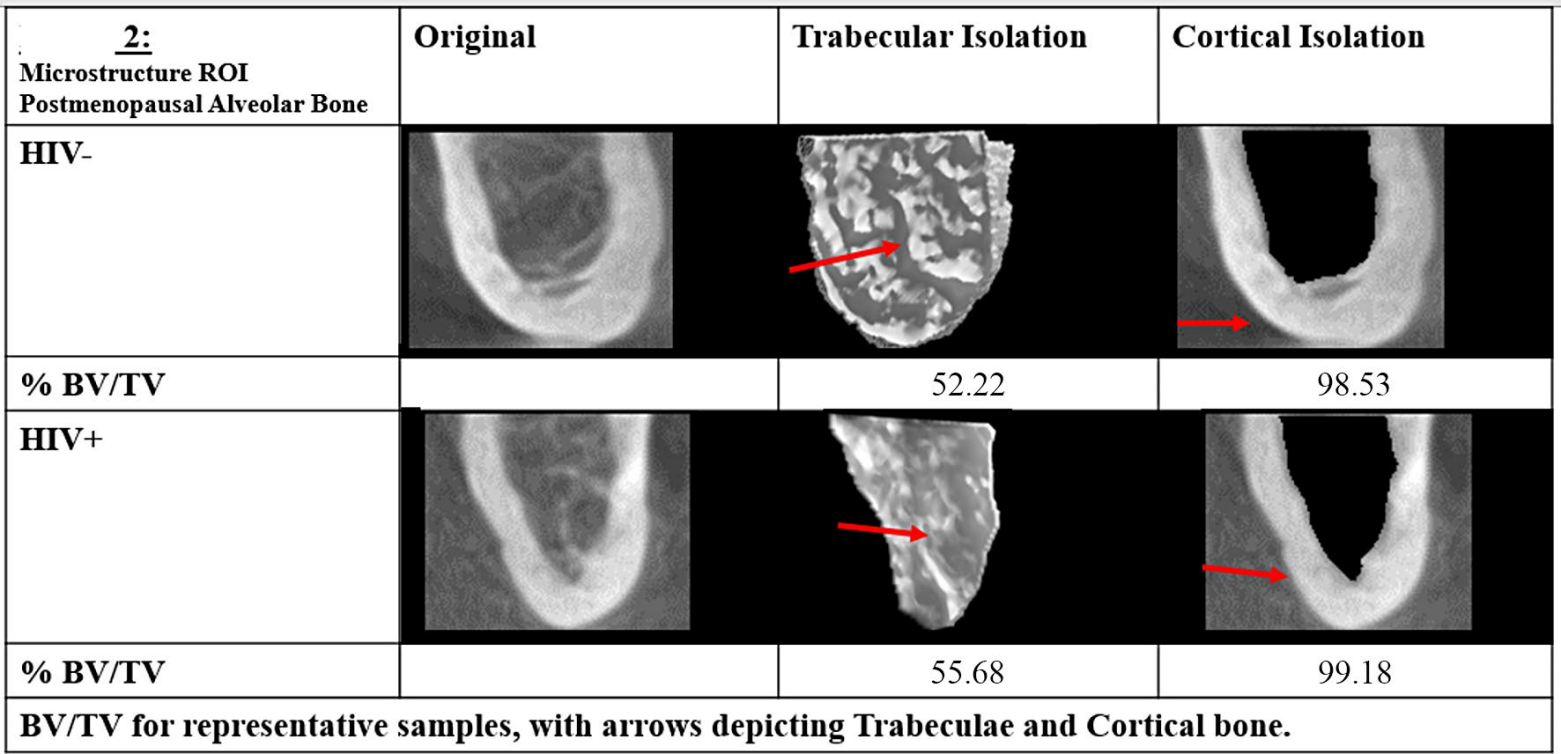
Representative Cone Beam 3-D images of the original mandibular alveolar bone and the trabecular and cortical compartments from people with HIV (PWH) and HIV-negative controls

Three-dimensional CBCT analysis of the microarchitecture of the alveolar bone surrounding the mental foramen region of the mandible revealed that women with HIV had significantly greater trabecular thickness (HIV + 7.25+/-1.25, HIV- 6.24+/-1.78; p < 0.001), lower trabecular number (HIV + 0.08+/-0.01, HIV- 0.09+/-0.02; p = 0.004), greater trabecular separation (HIV + 9.23+/-3.11, HIV- 7.99+/-3.23; p = 0.04), greater cortical BV/TV (HIV + 99.18+/-1.38, HIV- 98.53+/-1.8; p = 0.04), and lower cortical porosity (HIV + 0.82+/-1.38, HIV- 1.47+/-1.8; p = 0.04) compared to women without HIV (Table 1; Fig. 2).

### Multivariate logistic regression analysis on sub-cohort

**Fig. 3:**
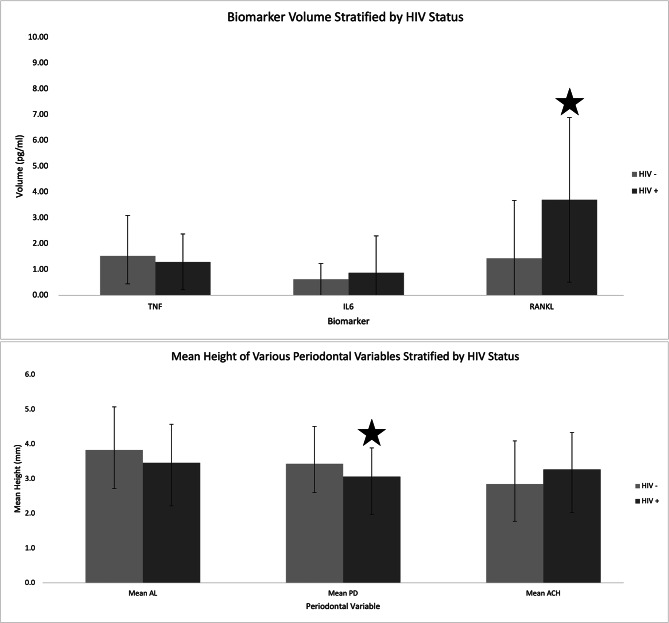
Gingival crevicular fluid biomarker of bone resorption cytokine levels (TNF a, IL-6, and RANKL), and Periodontal (Atachment Loss (AL), Probing Depth (PD) and Alveolar Crestal Height (ACH)) Variables on the subcohort of post-menopausal women with and without HIV (n = 114) excluding 12 white participants and 9 participants missing smoking and/or diabetes status from the full cohort. * Siginifiant difference p < 0.05 between HIV + vs. HIV-negative controls

Multivariate logistic regression was performed on a sub-cohort. The 12 white participants without HIV and nine other participants with missing diabetes and/ or smoking status were not included in this analysis, resulting in a total of 114 sub-cohort participants from 135 total participants. In an unadjusted analysis of the sub-cohort, RANKL (p = 0.001), mean PD (p = 0.045), number of teeth present (p = 0.002), trabecular thickness (p = 0.024), trabecular number (p = 0.015), cortical BV/TV (p = 0.038), and cortical porosity (p = 0.038) were significantly all different between women with and without HIV (Fig. 3). After adjusting for age, race/ethnicity (black/Hispanic), smoking status, and diabetes, RANKL (p < 0.0001), mean PD (p = 0.017), number of teeth present (p = 0.012), trabecular number (p = 0.009), and trabecular separation (p = 0.044) remained significant.

## Discussion

The effects of HIV infection on the alveolar bone and periodontal disease in women who have undergone the menopause transition is unknown. Therfore in this study we examined the alveolar bone microarchitecture by cone beam tomography, assessed gingival crevicular fluid cytokines and perfomed a periodontal examination in postmenopausal women with and without HIV. We found similar to other studies [4] that there was no difference in periodontal disease activity (BOP and CAL) in postmenopasal women with and without HIV. However, we did find that postmenopausal women with HIV in our study have on average four to five fewer teeth present than women without HIV.

In contrast, in an older oral substudy of the Women’s Interagency HIV Study (WIHS), they found that women with HIV had increased attachment loss, increased pocket depth and one fewer tooth present compared to women without HIV. [21, 22] The difference in the results between our study and the WIHS-Oral substudy could be attributed to age and menopausal status. The average age of participants in our study was 55 years old, whereas the average age in the WIHS-oral substudy was 37 years old at baseline. [22] Since the average age of menopause is 50 years old [23], it could be suggested that the menopause transition potentiates periodontal disease [24] in PWH. This may cause the teeth with periodontal disease to be extracted during the menopause transition in women with HIV resulting in less teeth present but better average attachment loss in postmenopausal women with HIV.

 [14 After an adjusted analysis in our study, we found that postemenopasal women with HIV had a decrease in trabecular number and an increase in trabecular spacing compared to postmenopausal women without HIV. Although the association between alveolar bone microarchitecture and tooth loss, periodontal disease, or dental implant survival is not well-defined [25–27], decreased trabecular number and increased trabecular spacing at the spine and radius have been shown to increase fracture risk. [28] It can be suggested that these parameters produce a similar mechanism in alveolar bone, but future longitudinal studies are needed to determine any such relationships.

This study found that after an adjusted analysis, GCF RANKL levels remained significantly higher among in women with HIV. RANKL is the major cytokine involved in periodontal disease-associated alveolar bone resorption. [29] We have previously found that the oral microbiome in postmenopausal women with HIV with severe periodontal disease was enriched with bacteria harboring lipopolysaccharides (LPS) compared to postmenopausal women with HIV without severe periodontal disease. [30] LPS are believed to play a major role in mediating periodontal disease-associated alveolar bone loss by in part increasing RANKL expression. [31] Therefore, it could be suggested that the increased RANKL levels seen in HIV infection contribute to alveolar bone deterioration seen in PWH.

Another explanation for fewer teeth among PWH is decreased dental care utilization, 19% of women with HIV in the US reported unmet dental needs [32] as a result of bias and/or barriers felt in seeking oral healthcare. Recent studies have shown that the majority of dentists are still uncomfortable providing dental care PWH, which may delay care and treatment. [33] PWH also continue to report high levels of stigmatizing and discriminatory attitudes and behavior in the dental setting, which were strongly associated with the avoidance of dental care. [34] The results of this study add to the literature a better understanding of the impact of aging and menopause on PWH, and effects on alveolar bone. It brings to light the need for PWH to have greater access to regular dental care in order for this vulnerable population to be better served by the medical community.

The World Health Organization has identified that keeping a functional, esthetic, and natural dentition of 21 or more teeth during one’s lifetime should be oral health treatment goal for everyone. [35] In our study we found that middle aged women with HIV living in New York city had on average < 18 teeth present. It is generally accepted that People living with HIV on Antiretroviral therapy have accelerated biological aging. [36, 37] In a recent review, it has been suggested that the characteristics of biological aging-cellular senescence, stem cell exhausation and immunoaging are also involved in maintaining periodontal homeostasis leading to increased tooth loss in subjects whose biological age at baseline is higher than their chronological age. [38] Other studies have shown that as people with HIV get older they are more likely to develop moderate to severe periodontal disease [39] and have increased tooth loss. [40] Therefore in order to maintain a functional dentition (> 20 teeth present) in peole with HIV throughout their lifetime, it is important to aggressively treat periodontal disease earlier to prevent future tooth loss as they potentially undergo accelerated biological cellular aging in the periodontal complex.

## Conclusion

Postmenopausal women with HIV have higher GCF RANKL levels and deterioration of the alveolar trabecular bone microarchitecture that may contribute to the observed greater tooth loss.

### Limitations

The sample size of the study was small which makes it difficult to extrapolate our data to the entire PWH population. Postmenopausal status was self-reported and not confirmed by longitudinal estradial levels, so there is a chance of misclassification, especially in people under the age of 40. The race/ethnicity of recruited participants was biased and more representative of people attending a New York City HIV clinic and dental clinic than the general population.

## Data Availability

The datasets generated and/or analysed during the current study are not publicly available due to protected health information but de-identified data are available from the corresponding author on reasonable request.
